# Impact of breastfeeding on risk of glucose intolerance in early postpartum after gestational diabetes

**DOI:** 10.3389/fendo.2024.1374682

**Published:** 2024-06-12

**Authors:** Yana Vanlaer, Caro Minschart, Hannah Vrolijk, Paul Van Crombrugge, Carolien Moyson, Johan Verhaeghe, Roland Devlieger, Sofie Vandeginste, Hilde Verlaenen, Chris Vercammen, Toon Maes, Els Dufraimont, Nele Roggen, Christophe De Block, Yves Jacquemyn, Farah Mekahli, Katrien De Clippel, Annick Van Den Bruel, Anne Loccufier, Inge Van Pottelbergh, Nele Myngheer, Pascale Abrams, Wouter Vinck, Liesbeth Leuridan, Sabien Driessens, Jaak Billen, Christophe Matthys, Annick Bogaerts, Annouschka Laenen, Chantal Mathieu, Katrien Benhalima

**Affiliations:** ^1^ Clinical and Experimental Endocrinology, Department of Chronic Diseases and Metabolism, KU Leuven, Leuven, Belgium; ^2^ Faculty of Medicine, KU Leuven, Leuven, Belgium; ^3^ Department of Endocrinology, OLV Ziekenhuis Aalst-Asse-Ninove, Aalst, Belgium; ^4^ Department of Obstetrics & Gynecology, UZ Gasthuisberg, KU Leuven, Leuven, Belgium; ^5^ REALIFE Research Group, Research Unit Woman and Child, Department of Development and Regeneration, KU Leuven, Leuven, Belgium; ^6^ Department of Obstetrics and Gynecology, GZA Hospitals Sint-Augustinus, Antwerp, Belgium; ^7^ Department of Obstetrics & Gynecology, OLV Ziekenhuis Aalst-Asse-Ninove, Aalst, Belgium; ^8^ Department of Endocrinology, Imelda Ziekenhuis, Bonheiden, Belgium; ^9^ Department of Obstetrics & Gynecology, Imelda Ziekenhuis, Bonheiden, Belgium; ^10^ Department of Endocrinology-Diabetology-Metabolism, Antwerp University Hospital, Edegem, Belgium; ^11^ Department of Obstetrics & Gynecology, Antwerp University Hospital, Edegem, Belgium; ^12^ ASTARC and GHI, Antwerp University, Antwerp, Belgium; ^13^ Department of Endocrinology, Kliniek St-Jan Brussel, Brussel, Belgium; ^14^ Department of Obstetrics & Gynecology, Kliniek St-Jan Brussel, Brussel, Belgium; ^15^ Department of Endocrinology, AZ St Jan Brugge, Brugge, Belgium; ^16^ Department of Obstetrics & Gynecology, AZ St Jan Brugge, Brugge, Belgium; ^17^ Department of Endocrinology, OLV Hospital Aalst, Aalst, Belgium; ^18^ Department of Endocrinology, General Hospital Groeninge Kortrijk, Kortrijk, Belgium; ^19^ Department of Endocrinology, ZAS Hospital Sint-Vincentius, Antwerpen, Belgium; ^20^ Department of Endocrinology, ZAS Hospital Sint-Augustinus, Antwerpen, Belgium; ^21^ Department of Endocrinology, General Hospital Klina, Brasschaat, Belgium; ^22^ Department of Laboratory Medicine, University Hospitals Leuven, Leuven, Belgium; ^23^ Department of Endocrinology, University Hospitals Leuven, Leuven, Belgium; ^24^ Department of Development and Regeneration, KU Leuven, Leuven, Belgium; ^25^ Faculty of Health, University of Plymouth, Plymouth, Devon, United Kingdom; ^26^ Center of Biostatics and Statistical Bioinformatics, KU Leuven, Leuven, Belgium

**Keywords:** breastfeeding, mixed milk feeding, postpartum glucose intolerance, gestational diabetes mellitus, impaired beta-cell function, insulin resistance

## Abstract

**Aims:**

To determine the impact of breastfeeding on the risk of postpartum glucose intolerance in women with gestational diabetes.

**Methods:**

Sub-analysis of two multi-centric prospective cohort studies (BEDIP-N and MELINDA) in 1008 women with gestational diabetes. Data were collected during pregnancy and at a mean of 12 weeks postpartum. Multivariate logistic regression was used to estimate the effect of breastfeeding on glucose intolerance, with adjustment for ethnicity, education, income, professional activity and BMI.

**Results:**

Of all participants, 56.3% (567) breastfed exclusively, 10.1% (102) gave mixed milk feeding and 33.6% (339) did not breastfeed. Mean breastfeeding duration was 3.8 ± 2.4 and 3.7 ± 2.1 months in the breastfeeding and mixed milk feeding groups (p=0.496). The rate of glucose intolerance was lower in both the breastfeeding [22.3% (126)] and mixed milk feeding [25.5% (26)] groups compared to the no breastfeeding group [29.5% (100)], with an adjusted OR of 0.7 (95% CI 0.5–1.0) for glucose intolerance in the breastfeeding group compared to no breastfeeding group and an adjusted OR of 0.7 (95% CI 0.4–1.2) for the mixed milk feeding group compared to the no breastfeeding group. Postpartum, breastfeeding women had a lower BMI, less often postpartum weight retention, lower fasting triglycerides, less insulin resistance and a higher insulin secretion-sensitivity index-2 than the mixed milk feeding and no breastfeeding group. The mixed milk feeding group was more often from an non-White background, had a lower blood pressure and lower fasting triglycerides compared to the no breastfeeding group.

**Conclusions:**

Breastfeeding (exclusive and mixed milk feeding) is associated with less glucose intolerance and a better metabolic profile in early postpartum in women with gestational diabetes.

## 1 Introduction

Women with gestational diabetes mellitus (GDM), defined as “diabetes diagnosed during pregnancy, provided that overt diabetes has been excluded in early pregnancy” (1), are more likely to develop type 2 diabetes mellitus (T2DM) postpartum (2). Around 30–50% of all women with GDM develop T2DM within 10 years after their pregnancy (3–5). Previous studies have shown that T2DM can be prevented in this population, by adopting changes in lifestyle and/or by medication (6, 7). However, adherence to a healthy lifestyle is often low in early postpartum due to barriers such as lack of time, need for childcare and lack of social support (8). Besides lifestyle changes, lactation has also shown to reduce the risk to develop T2DM (9). The World Health Organization (WHO) and United Nations International Childrens Emergency Fund (UNICEF) recommend exclusive BF for the first six months after childbirth, since breastfeeding (BF) has known benefits for the child (10). A protective effect of BF, is particularly seen when women BF for a longer duration (at least six months) (11, 12). Two large cohort studies have demonstrated a protective effect of lactation on the evolution of GDM to T2DM (9, 13). BF is associated with lower fasting glucose levels and improved insulin sensitivity (14).

The evidence for the effect of BF on glucose intolerance in the early postpartum period in women with GDM remains incomplete (14). Limitations of previous research include small sample sizes, inclusion of only women with overweight or obesity and distinguished only between exclusive BF or exclusive formula feeding. Women who gave mixed milk feeding (MMF) were not included in previous studies (15–17). We investigated therefore the risk of glucose intolerance in early postpartum in a *post-hoc* analysis of two large existing cohorts of women with a recent history of GDM, comparing women with exclusive BF, women who gave MMF and women who did not breastfeed (NBF).

## 2 Subjects and methods

### 2.1 Study design and setting

This study was a sub-analysis of the ‘Belgian Diabetes in Pregnancy study’ (BEDIP-N) (NCT02036619) and ‘Mobile-Based Lifestyle Intervention in Women with Glucose Intolerance after Gestational Diabetes Mellitus study’ (MELINDA) (NCT03559621). Both studies were published previously (18, 19). The BEDIP-N study was a large Belgian multi-centric prospective cohort study from 2014–2018 (18). This study enrolled 2006 pregnant women in early pregnancy to evaluate the diagnostic accuracy of different screening strategies for GDM based on the ‘International Association of Diabetes and Pregnancy Study Groups’ (IADPSG) criteria (20). All women without (pre)diabetes received universal screening for GDM between 24–28 weeks of pregnancy with a 75g 2-hour OGTT.

The Melinda study (performed between 2019–2023) was a multicenter randomized controlled trial (RCT), to investigate the efficacy and feasibility of a blended-care, telephone- and mobile-based lifestyle intervention to reach weight goals in 240 women with prediabetes after a recent history of GDM (diagnosed with the IADPSG criteria) (19, 20). In the Melinda study, baseline data were collected at 3 months postpartum of 1201 women with GDM who attended the postpartum OGTT (19). For this *post-hoc* analysis, only the baseline data were used.

In both studies, the American Diabetes Association (ADA)-recommended glycemic targets were used for treatment of GDM (21). If targets were not reached within two weeks after the start of lifestyle measures, treatment with insulin was started. Women with GDM received an invitation for a postpartum 75g OGTT 6–16 weeks after delivery. Glucose intolerance postpartum was defined as T2DM or prediabetes [defined as impaired fasting glucose (IFG; 100–125 mg/dL) and/or impaired glucose tolerance (IGT; 2-h glucose value on the OGTT between 140–199 mg/dL) or both] according to the ADA criteria (22).

Both studies received approval by the Institutional Review Boards of all participating centers and all investigations have been carried out in accordance with the principles of the Declaration of Helsinki as revised in 2008. Participants gave written informed consent prior to any trial-related activity.

### 2.2 Study visits and measurements

For both studies, baseline characteristics were collected for all eligible women through a clinical examination, self-administered questionnaires, collection of blood samples and extraction of data on medical history and pregnancy from the electronic medical records. At a mean of 12 weeks postpartum, a 75 g OGTT was performed with blood samples taken fasting and at 30, 60 and 120 min. Several self-administered questionnaires were completed by the participants. There was a self-designed questionnaire on general habits and socio-economic factors (18). The Food Frequency Questionnaire (FFQ) surveyed the frequency and portion size of consumption of foods and beverages (23). The International Physical Activity Questionnaire (IPAQ) measured physical activity such as job-related physical activity, transportation, house work and caring for family, recreation and time spent sitting (18, 24). The Center for Epidemiologic Studies-Depression (CES-D) questionnaire (widely used in pregnant and postpartum women) to assess symptoms of clinical depression over the past seven days (25). In both studies, the same self-designed questionnaire on BF and contraception was used, to collect information on the duration and frequency of BF. Women had to indicate what applied the most to them: [exclusive breastfeeding (< 45 ml formula feeding/day), half breastfeeding half formula feeding, or exclusive formula feeding (≥ 150ml formula feeding/day)] as well as on the type of contraception used (18). As the aim of this sub-analysis was to evaluate different degrees of intensity of BF, we only included women with a history of GDM, who received a postpartum OGTT and had data available on type of BF (exclusive, MMF or NBF) and on the duration of BF.

### 2.3 Procedures

In line with normal routine, GDM was diagnosed between 24–28 weeks of pregnancy with a 75g 2-hour OGTT using the IADPSG criteria. Women who were diagnosed with GDM, were reevaluated at a mean of 12 weeks postpartum with a 2-h 75 g OGTT to screen for glucose intolerance. The 2-h 75 g OGTT consisted of measurements of glucose and insulin at fasting, 30 min, 60 min and 120 min. At the time of the OGTT, a fasting lipid profile (total cholesterol, triglycerides, HDL and LDL cholesterol) and HbA1c were also measured. Participants were instructed to fast for at least 10 h and not to smoke nor engage in any physical activity. They were also instructed to drink only water, but no coffee, cola or any drink containing sugar or caffeine. The analyses of glucose (fasting, 30 min, 60 min and 120 min in fluoride-containing tubes) were performed locally (and sent to the lab immediately after collection) so that there was no delay in diagnosing (pre)diabetes. The blood samples for the analyses of lipid profile, HbA1c and insulin, were analyzed centrally at the laboratory of Leuven University Hospital (UZ Leuven) to ensure uniformity. Plasma glucose was measured by an automated colorimetric-enzymatic method on a Hitachi/Roche-Modular P analyzer (Basel, Switzerland). Insulin was measured by the immunometric ECLIA (Roche Modular E170). HbA1c was measured by Tosoh Automated Glycohemoglobin Analyzer HLC-723G8. Lipid levels were measured by the immunoassay analyzer Cobas 8000 (Roche, Basel, Switzerland). Coefficients of variance are 1% for glucose, 6% for insulin, about 2% for lipids and 2% for HbA1c in the Lab of UZ Leuven (18, 19).

Different indices of insulin sensitivity [the Matsuda index, a well-established measure of whole-body insulin sensitivity and the homeostasis model assessment of insulin resistance (HOMA-IR), a measure of largely hepatic insulin resistance] and β-cell function [HOMA-B, the insulinogenic index divided by HOMA-IR and the insulin secretion-sensitivity index-2 (ISSI-2), an OGTT-derived measure that is analogous to the disposition index obtained from the frequently sampled intravenous glucose tolerance test], were measured, as previously described (18, 19).

### 2.4 Pregnancy and delivery outcome data

The following pregnancy outcome data were collected: parity and pre-pregnancy BMI was stratified into underweight (BMI < 18.5 kg/m²), normal weight (BMI 18.5–24.9 kg/m²), overweight (BMI 25–29.9 kg/m²), and obesity (BMI ≥ 30 kg/m²). Obesity was further subdivided into class I (BMI 30–34.9 kg/m²), class II (35–39.9 kg/m²) and class III (BMI ≥ 40 kg/m²). Early postpartum weight retention was defined as the difference in weight measured at the postpartum OGTT and the pre-pregnancy weight (self-reported weight up to 1 month before pregnancy or weight measured during first prenatal consultation). Other data collected include birth weight, length, macrosomia (>4 kg), birth weight ≥4.5 kg, Large-for-gestational age (LGA) defined as birth weight >90 percentile according to standardized Flemish birth charts adjusted for sex of the baby and parity (26), small-for-gestational age (SGA) defined as birth weight <10 percentile according to standardized Flemish birth charts adjusted for sex of the baby and parity (26), and admission on the neonatal intensive care unit (NICU) (18). In line with normal routine in each center, admission to the NICU was decided by the neonatologist. The difference in weight between first prenatal visit and the time of the OGTT was calculated as early weight gain. The total gestational weight gain (GWG) was calculated as the difference in weight between first prenatal visit and the delivery. Excessive total GWG and inadequate total GWG were defined according to the National Academy of Medicine (NAM) guidelines, previously known as Institute of Medicine (IOM) (27).

### 2.5 Statistical analysis

Descriptive statistics were presented as frequencies and percentages for categorical variables, and means with standard deviations or medians with interquartile range for continuous variables. Group comparisons were performed using the Mann-Whitney U test for continuous or ordinal variables, and Chi square test or Fisher exact test in case of low (<5) cell frequencies for categorical variables.

Logistic regression was used for estimating the effect of BF on glucose intolerance, with correction for the following confounders: ethnicity, education, income, professional activity and pre-pregnancy body mass index (BMI). Adjustment was performed for baseline characteristics for which group differences were observed. Results were presented as odds ratios with 95% confidence intervals. All tests were performed at a 5% two-sided significance level. Analyses were performed by statistician A. Laenen by using SAS software (version 9.4 of the SAS System for Windows, 2023).

## 3 Results

**Figure 1:**
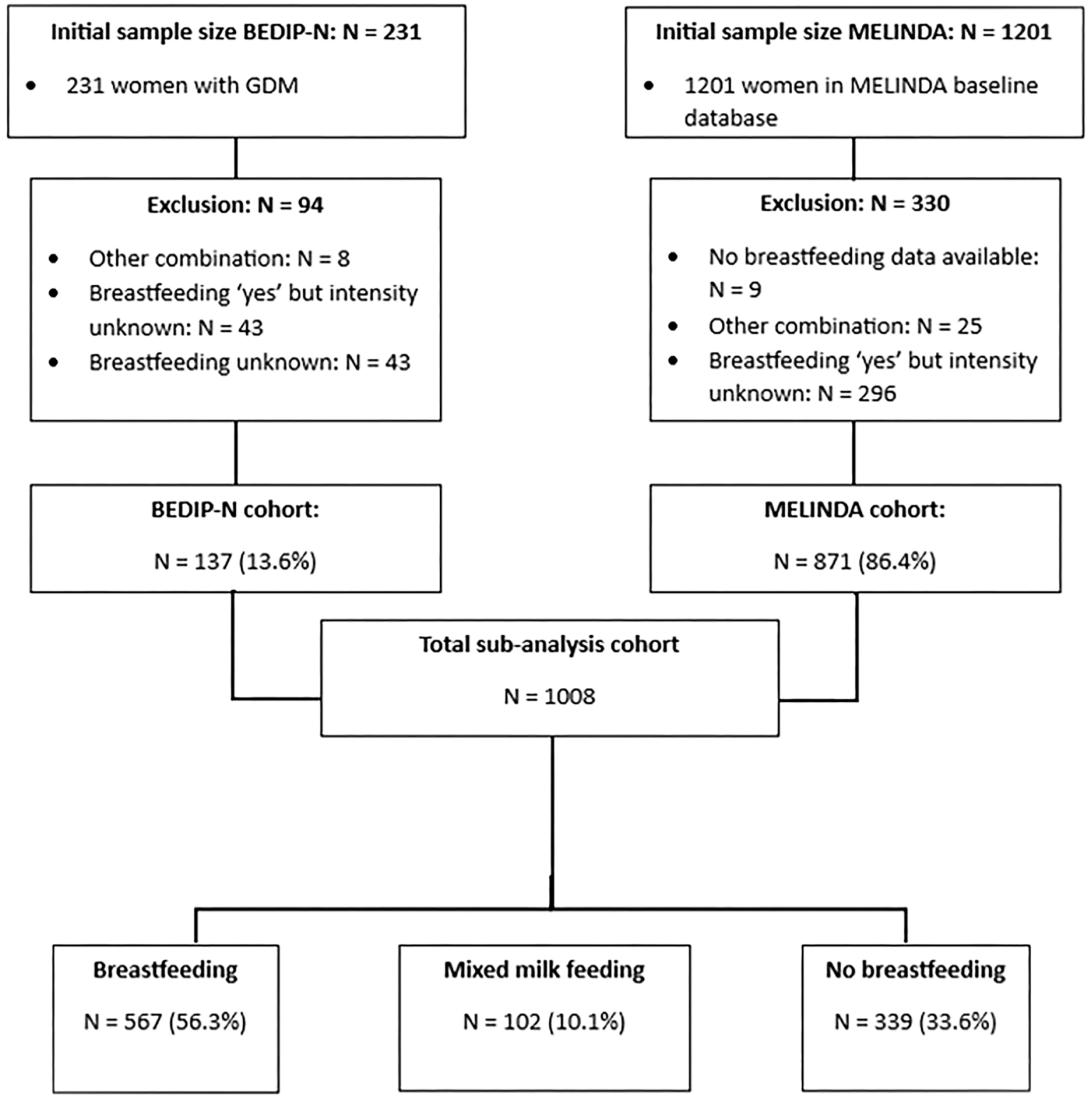
Flow chart of the total cohort included in the sub-analysis.

Women without data on whether they gave breastfeeding (N=43) or without data on the duration of BF (N=339), or women indicating a different intensity of BF other than exclusive BF, MMF or NBF (N=33), were excluded [a total of 415 women (29.2%)]. Women excluded from this analyses had in general similar characteristics compared to women included in this study, except for lower rates of multiparity and a slightly higher BMI (**Supplementary Table 3**). In total, data from 1008 women were included in this sub-analysis (**Figure 1**, **Supplementary Figures 1, 2**). Of all participants from both cohorts, 56.3% (567) gave BF exclusively, 10.1% (102) gave MMF, and 33.6% (339) did NBF at a mean of 12 weeks postpartum (**Figure 1**). Mean breastfeeding duration was respectively 3.8 ± 2.4 and 3.7 ± 2.1 months in the BF and MMF groups (**Table 1**).

**Table 1:** Participant postpartum characteristics according to breastfeeding behavior.

Total cohort (N=1008)							
	1. Breastfeeding (N=567, 56.3%)	2. Mixed milk feeding (N=102, 10.1%)	3. No breastfeeding (N=339, 33.6%)	Pairwise comparisons			
				p-value	1 vs 2	1 vs 3	2 vs 3
Postpartum							
Mean breastfeeding duration (months)	3.8 ± 2.4	3.7 ± 2.1	NA	0.635	0.492	NA	NA
% glucose intolerance	22.3 (126)	25.5 (26)	29.5 (100)	**0.004**	0.098	**0.019**	**0.011**
% Use of contraception	60.8 (345)	53.9 (55)	67.0 (227)	**0.035**	0.189	0.065	**0.016**
Timing OGTT (weeks)	11.9 ± 3.4	12.4 ± 3.7	12.0 ± 3.3	0.407	0.213	0.443	0.461
Weight mother (kg)	70.2 ± 13.7	73.8 ± 15.8	75.5 ± 15.8	**<.001**	**0.025**	**<.001**	0.338
BMI (kg/m²)	25.5 ± 4.7	27.3 ± 5.7	27.4 ± 5.6	**<.001**	**0.001**	**<.001**	0.972
Mean systolic blood pressure (mmHg)	115.8 ± 11.4	115.7 ± 13.7	120.8 ± 12.5	**<.001**	0.908	**<.001**	**<.001**
Mean diastolic blood pressure (mmHg)	74.0 ± 9.3	74.0 ± 10.2	76.2 ± 9.9	**0.002**	0.926	**<.001**	**0.039**
Waist circumference (cm)	88.0 ± 11.6	91.4 ± 12.0	92.2 ± 12.6	**<.001**	**0.003**	**<.001**	0.767
PPWR (kg)	-0.3 ± 4.9	2.0 ± 5.7	1.6 ± 5.6	**<.001**	**<.001**	**<.001**	0.285
HbA1c (%)	5.3 ± 0.3	5.4 ± 0.4	5.2 ± 0.3	**<.001**	**0.009**	**<.001**	**<.001**
Fasting total cholesterol (mg/dl) Fasting HDL-cholesterol (mg/dl) Fasting LDL-cholesterol (mg/dl) Fasting Triglycerides (mg/dl)	187.2 ± 36.1 64.3 ± 14.5 111.8 ± 32.7 68.9 ± 31.5	190.6 ± 35.2 60.7 ± 14.6 112.9 ± 29.2 100.1 ± 96.6	185.4 ± 34.0 58.8 ± 15.2 110.6 ± 31.5 111.0 ± 51.3	0.634 **<.001** 0.818 **<.001**	0.399 **0.029** 0.643 **<.001**	0.816 **<.001** 0.761 **<.001**	0.346 0.160 0.527 **<.001**
FPG (mg/dl) Glycemia 30 min (mg/dl) Glycemia 60 min (mg/dl) Glycemia 120 min (mg/dl)	86.1 ± 8.2 147.7 ± 25.2 146.3 ± 36.7 114.8 ± 30.7	90.4 ± 9.6 150.8 ± 28.9 142.8 ± 41.3 114.9 ± 33.5	91.1 ± 8.5 145.0 ± 25.3 136.5 ± 33.9 114.6 ± 30.1	**<.001** 0.131 **<.001** 0.797	**<.001** 0.345 0.138 0.510	**<.001** 0.152 **<.001** 0.839	0.254 0.054 0.339 0.591
Fasting insulin (pmol/l) Insulin 30 min (pmol/l) Insulin 60 min (pmol/l) Insulin 120 min (pmol/l)	49.7 ± 33.4 382.0 ± 221.1 280.7 ± 375.5 341.9 ± 252.2	67.6 ± 40.1 495.9 ± 319.1 589.0 ± 418.2 425.0 ± 267.8	74.5 ± 45.4 489.7 ± 324.4 556.7 ± 371.1 487.5 ± 370.1	**<.001** **<.001** **<.001** **<.001**	**<.001** **<.001** **0.001** **<.001**	**<.001** **<.001** **<.001** **<.001**	0.124 0.808 0.678 0.299
Matsuda insulin sensitivity	5.5 (3.8-7.6)	4.1 (2.7-6.2)	4.0 (2.6-5.5)	**<.001**	**<.001**	**<.001**	0.576
HOMA-IR	1.3 (0.9-1.9)	1.9 (1.1-2.9)	2.0 (1.4-3.1)	**<.001**	**<.001**	**0.001**	0.114
HOMA-B	98.3 (72.1-140.6)	115.5 (81.9-175.4)	120.8 (91.0-172.0)	**<.001**	**0.016**	**<.001**	0.205
ISSI-2	2.0 (1.6-2.6)	2.0 (1.5-2.7)	1.8 (1.4-2.4)	**0.010**	0.960	**0.003**	0.087
Insulinogenic index/ HOMA-IR	0.3 (0.2-0.4)	0.2 (0.1-0.4)	0.2 (0.1-0.3)	**<.001**	0.273	**<.001**	0.132
IPAQ METs category at time of OGTT % Low % Moderate % High	10.5 (59) 45.5 (256) 44.0 (248)	13.0 (13) 46.0 (46) 41.0 (41)	6.9 (23) 47.1 (157) 45.9 (153)	0.311	0.711	0.200	0.144
% IPAQ category low	11.8 (66)	10.8 (11)	9.2 (31)	0.482	0.772	0.227	0.633
% Clinical depression ( ≥16 on CES-D questionnaire)	15.0 (85)	24.5 (25)	19.8 (67)	**0.028**	**0.017**	0.063	0.301

OGTT, oral glucose tolerance test ; BMI, body mass index; PPWR; postpartum weight retention; HDL-cholesterol, high density lipoprotein cholesterol; LDL-cholesterol, low density lipoprotein cholesterol; FPG; fasting plasma glucose; HOMA-IR , Homeostasis Model of Assessment – Insulin Resistance; HOMA-B, Homeostasis Model of Assessment – Beta-cell Function; ISSI-2, insulin secretion-sensitivity index-2; IPAQ, International Physical Activity Questionnaire; METs, metabolic equivalent of task [MET] minutes/week; CES-D, Center for Epidemiologic Studies Depression; NA, not applicable.Categorical variables are presented as frequencies %(n); continuous variables are presented as mean ± SD if normally distributed and as median ± IQR if not normally distributed; Differences are considered significant at p-value<0.05. Bold means a statistical significant value of p<0.05.

### 3.1 Participant general characteristics, medical history and pregnancy outcomes according to breastfeeding behavior

Compared to NBF women, the exclusive BF and MMF groups were significantly more often from an non-White background. In comparison with the NBF group, women who BF were significantly more often higher educated, had a significantly lower pre-pregnancy BMI, had significantly less often excessive GWG, but significantly more often inadequate low GWG. Furthermore, women who BF had also significantly less often excessive GWG compared to the MMF group (**Table 2** and **Supplementary Table 1**).

**Table 2:** Participant general characteristics, medical history and pregnancy outcomes according to breastfeeding behavior.

Total cohort (N=1008)							
	1. Breastfeeding (N=567, 56.3%)	2. Mixed milk feeding (N=102, 10.1%)	3. No breastfeeding (N=339, 33.6%)	Pairwise comparisons			
				p-value	1 vs 2	1 vs 3	2 vs 3
General characteristics							
Age (years)	32.4 ± 4.1	32.5 ± 5.1	31.9 ± 4.5	0.230	0.853	0.100	0.287
% Non-White	19.3 (109)	32.3 (33)	8.3 (28)	**<.001**	**0.003**	**<.001**	**<.001**
% Higher degree diploma	83.1 (466)	67.0 (67)	62.0 (206)	**<.001**	**<.001**	**<.001**	0.368
% Paid professional activity	89.7 (507)	80.4 (82)	90.2 (305)	**0.014**	**0.007**	0.808	**0.007**
Monthly net income family %<€1500 %€1500-5000 % >€5000	3.7 (21) 81.2 (457) 15.1 (85)	8.9 (9) 83.2 (84) 7.9 (8)	3.2 (11) 89.0 (301) 7.7 (26)	**<.001**	**0.016**	**0.004**	0.056
% Living without partner	12.5 (71)	15.8 (16)	18.1 (61)	0.071	0.365	**0.022**	0.601
% History of smoking	24.3 (134)	30.0 (30)	29.6 (90)	0.171	0.225	0.090	0.940
Medical history							
% Multiparity	49.4 (280)	43.1 (44)	49.6 (168)	0.481	0.245	0.959	0.255
% First degree family history of T2DM	26.1 (142)	29.9 (29)	23.6 (76)	0.428	0.443	0.404	0.210
% Second degree family history of T2DM	58.0 (279)	59.8 (52)	55.9 (151)	0.776	0.759	0.581	0.529
% History of GDM	19.5 (68)	13.5 (7)	18.7 (39)	0.583	0.299	0.811	0.379
% History of PCOS	5.1 (28)	5.0 (5)	3.4 (11)	0.503	0.970	0.248	0.469
% History of miscarriage	33.7 (191)	28.4 (29)	32.1 (109)	0.565	0.298	0.635	0.477
Pre-pregnancy BMI (kg/m²)	25.6 ± 5.0	26.7 ± 5.7	26.8 ± 5.5	**0.005**	0.097	**0.002**	0.766
Delivery data maternal outcomes							
Gestational age (weeks)	38.4 ± 1.6	38.4 ± 1.7	38.3 ± 1.4	0.091	0.665	**0.046**	0.115
Gestational weight gain (kg)	8.6 ± 5.2	9.9 ± 7.1	9.2 ± 6.0	0.068	**0.022**	0.298	0.149
% Inadequate weight gain	56.1 (293)	45.4 (45)	46.7 (147)	**0.012**	0.051	**0.008**	0.833
% Excessive weight gain	12.8 (67)	24.2 (24)	22.5 (71)	**<.001**	**0.003**	**<.001**	0.725
Delivery data neonatal outcomes							
Birth weight (g)	3293.46 ± 484.160	3216.85 ± 520.837	3286.28 ± 484.923	0.411	0.219	0.428	0.491
Birth length (cm)	50 ± 2.068	49 ± 2.337	50 ± 1.831	0.122	**0.045**	0.950	0.070
% LGA	11.46 (65)	7.84 (8)	12.39 (42)	0.448	0.280	0.676	0.204
% SGA	4.41 (25)	11.76 (12)	5.31 (18)	**0.011**	**0.003**	0.537	**0.023**
% NICU admission	24.29 (34)	40.63 (13)	28.21 (22)	0.174	0.061	0.525	0.204

T2DM, type 2 diabetes mellitus; GDM, gestational diabetes mellitus; PCOS, polycystic ovary syndrome; BMI, body mass index; LGA, Large for Gestational Age; SGA, Small for Gestational Age; NICU, Neonatal Intensive Care Unit. Categorical variables are presented as frequencies %(n); continuous variables are presented as mean ± SD if normally distributed and as median ± IQR if not normally distributed; Differences are considered significant at p-value<0.05. Bold means a statistical significant value of p<0.05.

### 3.2 Participant postpartum characteristics according to breastfeeding behavior

Compared to the MMF and NBF groups, women who BF exclusively had postpartum a significant lower BMI -and waist circumference, significantly less often postpartum weight retention, significantly lower fasting triglycerides, significantly less insulin resistance, a lower HOMA-B index [98.3 (72.1–140.6) vs. 115.5 (81.9–175.4), p=0.016; 98.3 (72.1–140.6) vs. 120.8 (91.0–172.0), p<0.001] but a higher ISSI-2 index [only significant higher compared to NBF group with respectively 2.0 (1.6–2.6) vs. 2.0 (1.5–2.7), p=0.960 for MMF; 2.0 (1.6–2.6) vs. 1.8 (1.4–2.4), p=0.003 for NBF] (**Table 1**). Compared to the NBF group, the BF and MMF groups had a significant lower systolic blood pressure (SBP), lower diastolic blood pressure (DBP) and lower fasting triglycerides at the postpartum OGTT (**Table 1** and **Supplementary Table 2**).

### 3.3 Analysis of glucose intolerance status by breastfeeding group

The rate of glucose intolerance was significantly lower in both the BF (22.3%, p=0.019) and MMF (25.5%, p=0.011) groups compared to the NBF group (29.5%) (**Table 1**). Multivariate logistic regression was used to estimate the effect of BF on glucose intolerance in early postpartum, with adjustment for ethnicity, education, income, professional activity and pre-pregnancy BMI. The risk for glucose intolerance remained significantly lower in exclusively BF women compared to NBF women [adjusted OR of 0.7 (95% CI 0.5–1.0, p=0.0399)] (**Table 3**). The risk for glucose intolerance in the MMF group was no longer significantly lower compared to the NBF group after adjustment [adjusted OR of 0.7 (95% CI 0.4–1.2, p=0.2399)].

**Table 3:** Analysis of glucose intolerance status by breastfeeding group.

	Unadjusted OR			Adjusted* OR		
Variable	N	Mean difference (95% CI)	P-value	N	Mean difference (95% CI)	P-value
No breastfeeding (=ref)	1008		0.0523	985		0.1098
Breastfeeding		0.7 (0.5; 0.9)	0.0152		0.7 (0.5; 1.0)	0.0399
Mixed milk feeding		0.8 (0.5; 1.4)	0.4324		0.7 (0.4; 1.2)	0.2399

OR >(<) 1 means higher (lower) risk of glucose intolerance for group compared to reference. OR, Odds Ratio; CI, Confidence Interval. Adjusted for ethnicity, education, income, professional activity and pre-pregnancy BMI.

## 4 Discussion

We show in a large cohort of more than 1000 women with a recent history of GDM that glucose intolerance was present in respectively 22.3% (BF), 25.5% (MMF) and 29.5% (NBF) of all participants, with a lower risk for glucose intolerance in the exclusively BF and MMF women compared to NBF women. However, the risk for glucose intolerance was no longer significantly lower in the MMF group after adjustment for confounders. Studies have previously demonstrated that breastfeeding is associated with improved insulin sensitivity and improved glucose tolerance (14). Breastfeeding is therefore an effective, low-cost intervention that can be easily applied after childbirth (15).

Our study provides novel data that also women who gave MMF had a lower rate of glucose intolerance compared to the NBF group, which suggests that even MMF might have a positive effect on the glucose levels. These data suggest therefore that when exclusive BF is not possible, MMF should also be stimulated in the first months of the postpartum period. However, after adjustment for confounders, the risk for glucose intolerance was no longer significantly lower in the MMF group compared to the NBF group. This might be due to the lower sample size in the MMF group and the lack of data on the long-term. Exclusive BF remains therefore preferable since the stronger association with a lower risk for glucose intolerance, as also demonstrated in our study. Research on the effects of MMF remain scarce. A previous study included only a small number of women who were undertaking mixed feeding; therefore a dose response relationship could not be established (17).

Our results also demonstrate that women who BF exclusively had lower glucose intolerance rates despite a lower HOMA-B index than the MMF and NBF groups (with also a lower non-significant HOMA-B in the MMF group compared to the NBF group). On the other hand, in our study the ISSI-2 index was higher in the BF group compared to the NBF group. ISSI-2 is in general considered to be a more accurate marker of β-cell function in women with GDM as it reflects an insulin resistance-adjusted insulin secretion (analogous to the disposition index obtained from the frequently sampled intravenous glucose tolerance test). Findings of a possible more impaired β-cell function (with lower HOMA-B) in women who exclusive BF, has also been reported in a prospective observational study in 106 mainly White women, between the 3rd and 5th month after the delivery (28). In this study, β-cell function was independently and negatively associated with BF and circulating prolactin concentrations. A recent meta-analysis confirmed that elevated prolactin was associated with lower beta-cell function and higher insulin sensitivity in the postpartum period. However, the direction of causality remains unclear (29). Lipolysis is also known to contribute to decreasing β-cell function (30). The pathophysiology of pregnancy and BF requires a certain β-cell plasticity, enabling adequate increase during pregnancy and immediate decrease in β-cell function postpartum. Reasons for not reaching this plasticity are mothers with obesity and high insulin resistance, but also lean mothers with impaired β-cell function because of genetic or aging reasons (28).

Compared to NBF women, BF and MMF women were more often from an non-White background. These results are consistent with results from previous studies indicating that non-White women had higher BF rates than women from a White origin (31). The BF group, compared to the NBF group, was more often higher educated, which is in line with previous studies that have shown that women with a Master’s degree (or higher) are more likely to start BF and also maintain BF for a longer period of time than mothers with primary studies (32–34). Pre-pregnancy BMI is also lower in our BF population compared to the NBF group. This can be explained by the fact that mothers with above-normal pre-pregnancy BMI are at increased risk of BF cessation (35). In addition, it has been demonstrated that women with underweight or obesity have significantly lower rates of BF initiation compared to women with normal pre-pregnancy BMI (36, 37). BF initiation in women with obesity may be lower due to a biological barrier, with a decreased hormonal response in lactation (38). A large population-based study from Florida, showed that women with overweight or obesity had a lower prolactin response to suckling. This can compromise the ability of women with overweight/obesity to produce milk and, over time, could lead to early cessation of lactation (36). In addition, also women with underweight were less likely to initiate breastfeeding (57, 58). Possible reasons might be maternal complications, insufficient milk supply, sucking problems and work resumption (39). It is therefore important to provide additional support to women with underweight or obesity to initiate and maintain breastfeeding.

Our results also showed lower fasting triglycerides in the MMF and BF group compared to the NBF group. This is in line with a study evaluating the lipid profile after 12 months postpartum among 79 predominantly Hispanic and socioeconomically disadvantaged women, indicating that each month increase in BF duration was associated with lower fasting glucose levels and lower triglycerides levels (17, 40). BF was also associated with less postpartum weight retention in our study, which has been confirmed in previous studies (41–43). BF may help mobilize fat stores built up during pregnancy, leading to weight loss, provided there is no compensatory increase in energy intake (44). Although BF may help some women lose weight, it cannot be generalized across all women who BF. This is due to the fact that women who BF often experience additional pressures, such as having to adapt to the needs of a newborn baby and recover from childbirth, difficulties in establishing feeding routines and baby sleep routines, which can affect the mother’s psychological health. A combination of these factors is likely to affect the ability to maintain a healthy lifestyle (45). More research is needed to assess the direct impact of BF on postpartum weight management on the long-term and to explore in-depth the reasons why not all women who BF lose weight (45–49).

Previous research has shown a decrease in both SBP and DBP during a BF session (50). Our study confirms these results, as there was also a decreased SBP and DBP in the MMF and exclusive BF groups compared to the NBF group. A study with over 400 Asian women, showed a lower SBP in BF women at one month postpartum compared with those using other feeding modalities (51). This suggests a direct effect of breastfeeding on maternal blood pressure. Animal studies suggest that oxytocin activates an “antistress” response that reduces cardiovascular stress reactivity, which may therefore lead to a lower BP (52). Studies in humans have shown that oxytocin, which is released when the baby starts suckling, has an anti-stress and blood pressure lowering effect (53–55).

A major strength of our study is that we used the data of two large multi-centric prospective cohort studies containing broad demographic, medical and obstetrical outcomes, as well as detailed postpartum characteristics. In addition, the same questionnaire was used to collected data on BF in both studies. The outcomes of this sub-analysis were adjusted for several confounding variables. Furthermore, we also included a large group of women with MMF, as the available data on the association with risk for glucose intolerance is very limited for this group. A first limitation of the study is that our population consisted mainly of White women. The results may therefore not to be applicable to other ethnic populations. Furthermore, this was a *post-hoc* analysis, with lack of longitudinal data, since data were only collected during pregnancy and at a mean of 12 weeks postpartum. A third limitation is that the questionnaire evaluating intensity and duration of breastfeeding was self-designed. Women had to indicate which of the three categories (BF, MMF or NBF) was most applicable to them. The degree of breastfeeding can however vary over time. We have no data on the exact dose of breastfeeding. In addition, for this analyses, we had to exclude women who did not have complete data on breastfeeding. However, the general characteristics of women not included in this analyses, were similar except for a lower rate of multiparity and slightly higher BMI compared to women included in the study.

In conclusion, our results indicate that exclusive BF and with a lesser degree also MMF, is associated with a lower risk of glucose intolerance in early postpartum in women with GDM. In addition, our results show that both BF and MMF are associated with a better metabolic profile in early postpartum. These data suggest therefore that when exclusive BF is not possible, MMF should also be stimulated in the first months postpartum. It is therefore important to support both BF and MMF by educating women already prenatally about the benefits of breastfeeding.

## Data availability statement

The datasets presented in this study can be found in online repositories. The names of the repository/repositories and accession number(s) can be found in the article/**Supplementary Material.**

## Ethics statement

The studies involving humans were approved by Ethics Committee Research UZ/KU Leuven. The studies were conducted in accordance with the local legislation and institutional requirements. The participants provided their written informed consent to participate in this study.

## Author contributions

YV: Writing – original draft, Writing – review & editing. CMi: Writing – review & editing. HV: Writing – review & editing. PVC: Writing – review & editing. CMo: Writing – review & editing. JV: Writing – review & editing. RD: Writing – review & editing. SV: Writing – review & editing. HV: Writing – review & editing. CV: Writing – review & editing. TM: Writing – review & editing. ED: Writing – review & editing. NR: Writing – review & editing. CDB: Writing – review & editing. YJ: Writing – review & editing. FM: Writing – review & editing. KDC: Writing – review & editing. AVDB: Writing – review & editing. ALo: Writing – review & editing. IVP: Writing – review & editing. NM: Writing – review & editing. PA: Writing – review & editing. WV: Writing – review & editing. LL: Writing – review & editing. SD: Writing – review & editing. JB: Writing – review & editing. ChrM: Writing – review & editing. AB: Writing – review & editing. AL: Formal analysis, Writing – review & editing. CM: Writing – review & editing. KB: Writing – review & editing.

## References

[1] American Diabetes Association Professional Practice Committee. 1. Improving care and promoting health in populations: standards of care in diabetes—2024. Diabetes Care (2024) 47(Supplement_1):S11–S19. doi: 10.2337/dc24-S001 38078573 PMC10725798

[2] Bellamy L Casas JP Hingorani AD Williams D. Type 2 diabetes mellitus after gestational diabetes: a systematic review and meta-analysis. Lancet. (2009) 373:1773–9. doi: 10.1016/S0140-6736(09)60731-5 19465232

[3] Benhalima K Leuridan L Calewaert P Devlieger R Verhaeghe J Mathieu C. Glucose intolerance after a recent history of gestational diabetes. Int J Endocrinol. (2014) 2014:1–9. doi: 10.1155/2014/727652 PMC4142274 25180037

[4] Inoue H Ishikawa K Takeda K Kobayashi A Kurita K Kumagai J. Postpartum risk of diabetes and predictive factors for glucose intolerance in East Asian women with gestational diabetes. Diabetes Res Clin Pract [Internet]. (2018) 140:1–8. doi: 10.1016/j.diabres.2018.03.031 29596944

[5] O’Shea E Awang MH Kgosidialwa O Tuthill A. Abnormal glucose tolerance in women with prior gestational diabetes mellitus: a 4-year follow-up study. Irish J Med Sci (1971 -) [Internet]. (2023) 192:641–8. doi: 10.1007/s11845-022-03005-x 35419723

[6] Tuomilehto J Lindström J Eriksson JG Valle TT Hämäläinen H Ilanne-Parikka P. Prevention of type 2 diabetes mellitus by changes in lifestyle among subjects with impaired glucose tolerance. New Engl J Med. (2001) 344:1343–50. doi: 10.1056/NEJM200105033441801 11333990

[7] Shubrook JH Chen W Lim A. Evidence for the prevention of type 2 diabetes mellitus. J Am Osteopathic Assoc. (2018) 118. doi: 10.7556/jaoa.2018.158 30398570

[8] Sharma M Purewal TS Fallows S Kennedy L. The low-risk perception of developing type 2 diabetes among women with a previous history of gestational diabetes: a qualitative study. Pract Diabetes. (2019) 36:15. doi: 10.1002/pdi.2204

[9] Much D Beyerlein A Roßbauer M Hummel S Ziegler AG. Beneficial effects of breastfeeding in women with gestational diabetes mellitus. Mol Metab. (2014) 3:284–92. doi: 10.1016/j.molmet.2014.01.002 PMC3986583 24749058

[10] Bollipo S Pagali D Korrapolu HB Rahman MA. The first golden hour of breastfeeding: where do we stand? Int J Contemp Pediatr. (2018) 6:27. doi: 10.18203/2349-3291.ijcp20184688

[11] Victora CG Bahl R Barros AJD França GVA Horton S Krasevec J. Breastfeeding in the 21st century: epidemiology, mechanisms, and lifelong effect. Lancet. (2016) 387:475–90. doi: 10.1016/S0140-6736(15)01024-7 26869575

[12] Gunderson EP Hurston SR Ning X Lo JC Crites Y Walton D. Lactation and progression to type 2 diabetes mellitus after gestational diabetes mellitus. Ann Intern Med. (2015) 163:889–98. doi: 10.7326/M15-0807 PMC5193135 26595611

[13] Ziegler AG Wallner M Kaiser I Rossbauer M Harsunen MH Lachmann L. Long-term protective effect of lactation on the development of type 2 diabetes in women with recent gestational diabetes mellitus. Diabetes. (2012) 61:3167–71. doi: 10.2337/db12-0393 PMC3501852 23069624

[14] Gunderson EP Hedderson MM Chiang V Crites Y Walton D Azevedo RA. Lactation intensity and postpartum maternal glucose tolerance and insulin resistance in women with recent GDM. Diabetes Care. (2012) 35:50–6. doi: 10.2337/dc11-1409 PMC3241296 22011407

[15] Dijigow FB Paganoti C de F da Costa RA Francisco RPV Zugaib M. Influência da amamentação nos resultados do teste oral de tolerância à glicose pós-parto de mulheres com diabetes mellitusgestacional. Rev Bras Ginecologia e Obstetrícia. (2015) 37:565–70. doi: 10.1590/SO100-720320150005488 26647845

[16] O’Reilly M Avalos G Dennedy MC O’Sullivan EP Dunne FP. Breast-feeding is associated with reduced postpartum maternal glucose intolerance after gestational diabetes. Ir Med J. (2012) 105. 22838108

[17] Shub A Miranda M Georgiou HM McCarthy EA Lappas M. The effect of breastfeeding on postpartum glucose tolerance and lipid profiles in women with gestational diabetes mellitus. Int Breastfeed J. (2019) 14:4. doi: 10.1186/s13006-019-0238-5 31708997 PMC6829979

[18] Benhalima K Van Crombrugge P Verhaeghe J Vandeginste S Verlaenen H Vercammen C. The Belgian Diabetes in Pregnancy Study (BEDIP-N), a multi-centric prospective cohort study on screening for diabetes in pregnancy and gestational diabetes: methodology and design. BMC Pregnancy Childbirth. (2014) 14:226. doi: 10.1186/1471-2393-14-226 25015413 PMC4227277

[19] Minschart C Maes T De Block C Van Pottelbergh I Myngheer N Abrams P. Mobile-based lifestyle intervention in women with glucose intolerance after gestational diabetes mellitus (MELINDA), A multicenter randomized controlled trial: methodology and design. J Clin Med. (2020) 9:2635. doi: 10.3390/jcm9082635 32823771 PMC7465345

[20] International Association of Diabetes and Pregnancy Study Groups. Recommendations on the diagnosis and classification of hyperglycemia in pregnancy. Diabetes Care. (2010) 33:676–82. doi: 10.2337/dc09-1848 PMC2827530 20190296

[21] ElSayed NA Aleppo G Aroda VR Bannuru RR Brown FM Bruemmer D. 6. Glycemic targets: standards of care in diabetes—2023. Diabetes Care. (2023) 46:S97–110. doi: 10.2337/dc23-S006 36507646 PMC9810469

[22] American Diabetes Association. Diagnosis and classification of diabetes mellitus. Diabetes Care. (2010) 33:S62–9. doi: 10.2337/dc10-S062 PMC2797383 20042775

[23] Matthys C Meulemans A Schueren B. Development and validation of general FFQ for use in clinical practice. Ann Nutr Metab. (2015) 67.

[24] Harrison CL Thompson RG Teede HJ Lombard CB. Measuring physical activity during pregnancy. Int J Behav Nutr Phys Activity. (2011) 8:19. doi: 10.1186/1479-5868-8-19 PMC3069935 21418609

[25] Dalfrà MG Nicolucci A Bisson T Bonsembiante B Lapolla A. Quality of life in pregnancy and post-partum: a study in diabetic patients. Qual Life Res. (2012) 21:291–8. doi: 10.1007/s11136-011-9940-5 21633879

[26] Devlieger H Martens G Bekaert A Eeckels R. Standaarden van geboortegewicht-voor-zwangerschapsduur voor de vlaamse boreling. Tijdschr Geneeskd. (2000) 56:1–14. doi: 10.47671/TVG.56.1.5000625

[27] Rasmussen KM Yaktine AL. Weight gain during pregnancy. Washington, D.C: National Academies Press (2009). Available at: https://pubmed.ncbi.nlm.nih.gov/20669500/. 20669500

[28] Harreiter J Vila G Leitner K Wattar L Leutner M Worda C. Decreased beta-cell function in breastfeeding obese and non-obese women: A prospective observational study. Clin Nutr. (2019) 38:2790–8. doi: 10.1016/j.clnu.2018.11.035 30583966

[29] Rassie K Giri R Joham AE Mousa A Teede H. Prolactin in relation to gestational diabetes and metabolic risk in pregnancy and postpartum: A systematic review and meta-analysis. Front Endocrinol (Lausanne). (2022) 13. doi: 10.3389/fendo.2022.1069625 PMC9813437 36619539

[30] DeFronzo RA. From the triumvirate to the ominous octet: A new paradigm for the treatment of type 2 diabetes mellitus. Diabetes. (2009) 58:773–95. doi: 10.2337/db09-9028 PMC2661582 19336687

[31] Griffiths LJ Tate AR Dezateux C. The contribution of parental and community ethnicity to breastfeeding practices: evidence from the Millennium Cohort Study. Int J Epidemiol. (2005) 34:1378–86. doi: 10.1093/ije/dyi162 16109734

[32] Haas DM Yang Z Parker CB Chung J Parry S Grobman WA. Factors associated with duration of breastfeeding in women giving birth for the first time. BMC Pregnancy Childbirth. (2022) 22:722. doi: 10.1186/s12884-022-05038-7 36138368 PMC9494803

[33] Lechosa-Muñiz C Paz-Zulueta M Sota SM de Adana Herrero MS del Rio EC Llorca J. Factors associated with duration of breastfeeding in Spain: a cohort study. Int Breastfeed J. (2020) 15:79. doi: 10.1186/s13006-020-00324-6 32907592 PMC7488233

[34] Levinienė G Tamulevičienė E Kudzytė J Petrauskienė A Zaborskis A Aželienė I. Factors associated with breastfeeding duration. Medicina (Kaunas). (2013) 49:415–21. doi: 10.3390/medicina49090065 24589578

[35] Martin H Thevenet-Morrison K Dozier A. Maternal pre-pregnancy body mass index, gestational weight gain and breastfeeding outcomes: a cross-sectional analysis. BMC Pregnancy Childbirth. (2020) 20:471. doi: 10.1186/s12884-020-03156-8 32807132 PMC7433137

[36] Thompson LA Zhang S Black E Das R Ryngaert M Sullivan S. The association of maternal pre-pregnancy body mass index with breastfeeding initiation. Matern Child Health J. (2013) 17:1842–51. doi: 10.1007/s10995-012-1204-7 23247667

[37] Turcksin R Bel S Galjaard S Devlieger R. Maternal obesity and breastfeeding intention, initiation, intensity and duration: a systematic review. Matern Child Nutr. (2014) 10:166–83. doi: 10.1111/j.1740-8709.2012.00439.x PMC6860286 22905677

[38] Rasmussen KM Kjolhede CL. Prepregnant overweight and obesity diminish the prolactin response to suckling in the first week postpartum. Pediatrics. (2004) 113:e465–71. doi: 10.1542/peds.113.5.e465 15121990

[39] Guelinckx I Devlieger R Bogaerts A Pauwels S Vansant G. The effect of pre-pregnancy BMI on intention, initiation and duration of breast-feeding. Public Health Nutr. (2012) 15:840–8. doi: 10.1017/S1368980011002667 22035605

[40] Niu Z Naya CH Reynaga L Toledo-Corral CM Johnson M Yang T. Association of breastfeeding duration with 12-month postpartum blood lipids in a predominately lower-income hispanic pregnancy cohort in los angeles. Int J Environ Res Public Health. (2022) 19:3008. doi: 10.3390/ijerph19053008 35270701 PMC8910591

[41] Baker JL Gamborg M Heitmann BL Lissner L Sørensen TI Rasmussen KM. Breastfeeding reduces postpartum weight retention. Am J Clin Nutr. (2008) 88:1543–51. doi: 10.3945/ajcn.2008.26379 19064514

[42] He X Zhu M Hu C Tao X Li Y Wang Q. Breast-feeding and postpartum weight retention: a systematic review and meta-analysis. Public Health Nutr. (2015) 18:3308–16. doi: 10.1017/S1368980015000828 PMC10271764 25895506

[43] Tahir MJ Haapala JL Foster LP Duncan KM Teague AM Kharbanda EO. Association of full breastfeeding duration with postpartum weight retention in a cohort of predominantly breastfeeding women. Nutrients. (2019) 11:938. doi: 10.3390/nu11040938 31027268 PMC6520964

[44] van Raaij J Schonk C Vermaat-Miedema S Peek M Hautvast J. Energy cost of lactation, and energy balances of well-nourished Dutch lactating women: reappraisal of the extra energy requirements of lactation. Am J Clin Nutr. (1991) 53:612–9. doi: 10.1093/ajcn/53.3.612 2000814

[45] Neville CE McKinley MC Holmes VA Spence D Woodside JV. The relationship between breastfeeding and postpartum weight change—a systematic review and critical evaluation. Int J Obes. (2014) 38:577–90. doi: 10.1038/ijo.2013.132 23892523

[46] Hollis JL Crozier SR Inskip HM Cooper C Godfrey KM Harvey NC. Modifiable risk factors of maternal postpartum weight retention: an analysis of their combined impact and potential opportunities for prevention. Int J Obes. (2017) 41:1091–8. doi: 10.1038/ijo.2017.78 PMC5500180 28337028

[47] Brandhagen M Lissner L Brantsaeter AL Meltzer HM Häggkvist AP Haugen M. Breast-feeding in relation to weight retention up to 36 months postpartum in the Norwegian Mother and Child Cohort Study: modification by socio-economic status? Public Health Nutr. (2014) 17:1514–23. doi: 10.1017/S1368980013001869 PMC10282327 23915637

[48] Martin JE Hure AJ Macdonald-Wicks L Smith R Collins CE. Predictors of post-partum weight retention in a prospective longitudinal study. Matern Child Nutr. (2014) 10:496–509. doi: 10.1111/j.1740-8709.2012.00437.x 22974518 PMC6860352

[49] Olson CM Strawderman MS Hinton PS Pearson TA. Gestational weight gain and postpartum behaviors associated with weight change from early pregnancy to 1 y postpartum. Int J Obes. (2003) 27:117–27. doi: 10.1038/sj.ijo.0802156 12532163

[50] Jonas W Nissen E Ransjö-Arvidson AB Wiklund I Henriksson P Uvnäs-Moberg K. Short- and long-term decrease of blood pressure in women during breastfeeding. Breastfeeding Med. (2008) 3:103–9. doi: 10.1089/bfm.2007.0031 18563998

[51] Kashiwakura I Ebina S. Influence of breastfeeding on maternal blood pressure at one month postpartum. Int J Womens Health. (2012) 333:334-7. doi: 10.2147/IJWH PMC3410704 22870047

[52] Groer MW Jevitt CM Sahebzamani F Beckstead JW Keefe DL. Breastfeeding status and maternal cardiovascular variables across the postpartum. J Womens Health (Larchmt). (2013) 22:453–9. doi: 10.1089/jwh.2012.3981 PMC3653385 23659484

[53] Grewen KM Light KC. Plasma oxytocin is related to lower cardiovascular and sympathetic reactivity to stress. Biol Psychol. (2011) 87:340–9. doi: 10.1016/j.biopsycho.2011.04.003 PMC3225916 21540072

[54] Heinrichs M Meinlschmidt G Neumann I Wagner S Kirschbaum C Ehlert U. Effects of suckling on hypothalamic-pituitary-adrenal axis responses to psychosocial stress in postpartum lactating women. J Clin Endocrinol Metab. (2001) 86:4798–804. doi: 10.1210/jcem.86.10.7919 11600543

[55] Light KC Smith TE Johns JM Brownley KA Hofheimer JA Amico JA. Oxytocin responsivity in mothers of infants: A preliminary study of relationships with blood pressure during laboratory stress and normal ambulatory activity. Health Psychol. (2000) 19:560–7. doi: 10.1037//0278-6133.19.6.560 11129359

