# Supplementary material for: Impact of breastfeeding on risk of glucose intolerance in early postpartum after gestational diabetes
Source: Front Endocrinol (Lausanne). 2024 Jun 12;15:1374682. doi: 10.3389/fendo.2024.1374682 (PMC11199774; doi:10.3389/fendo.2024.1374682)
Supplement: Supplementary file 1 [file DataSheet_1.pdf]

Supplementary Material

1 Supplementary Figures and Tables

1.1 Supplementary Figure

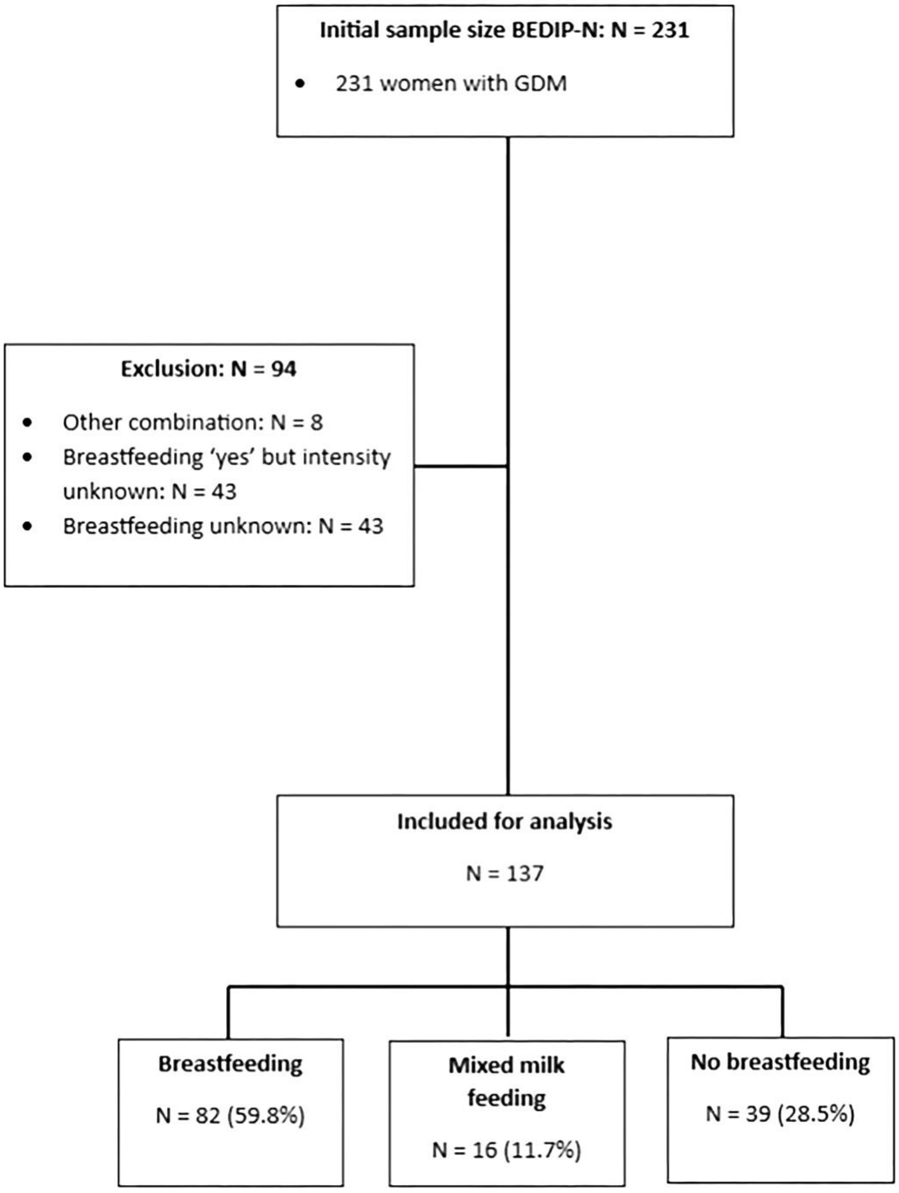

Supplementary Figure I. Flow chart of participants of the BEDIP-N study included in the sub-analysis.

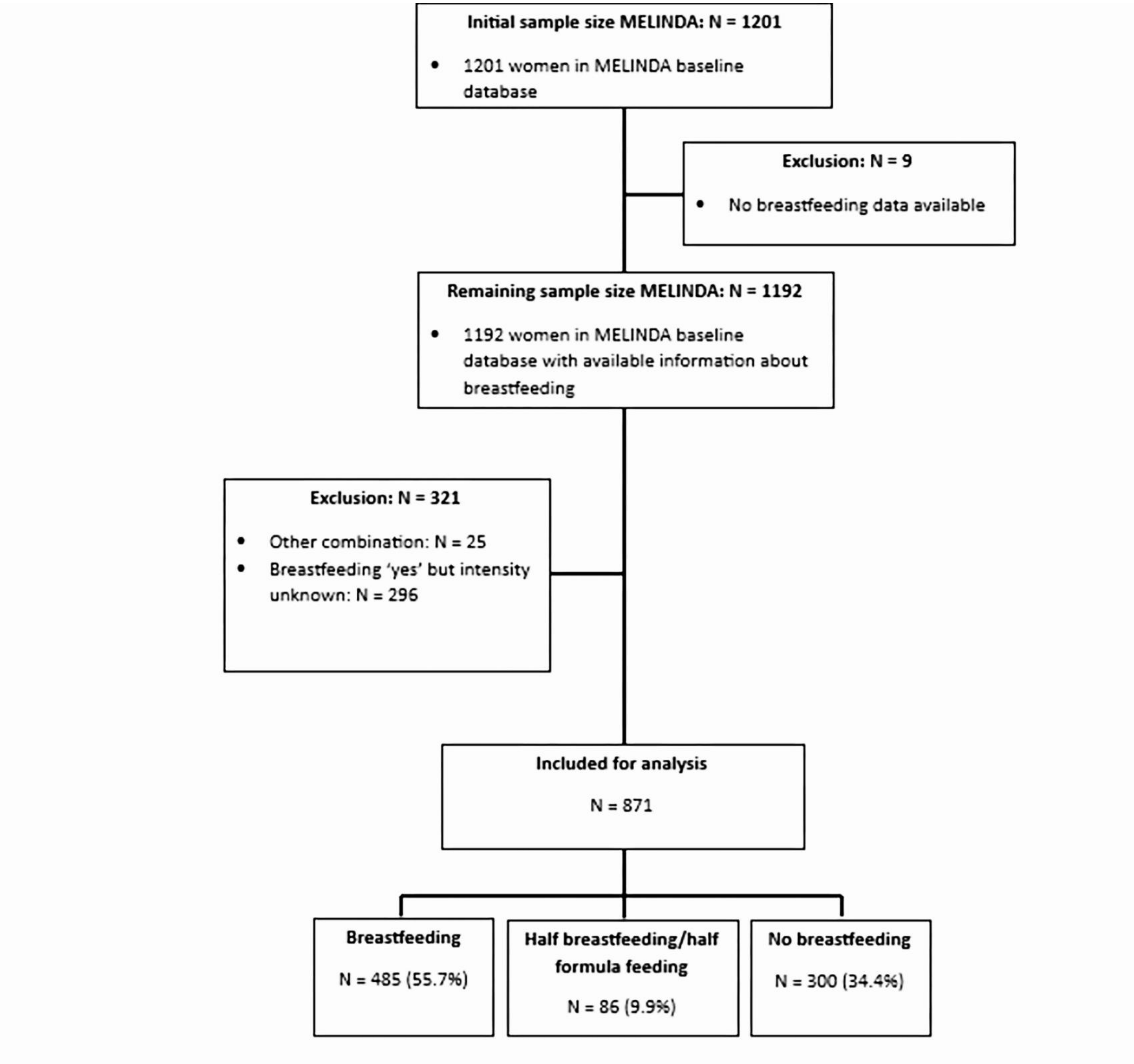

Supplementary Figure II. Flow chart of participants of the MELINDA study included in the sub-analysis.

1.2 Supplementary Tables

Supplementary Table I. Participant general characteristics, medical history and pregnancy outcomes according to breastfeeding behavior in the BEDIP-N and MEDLINA studies

Total cohort (N=1008)							
	**1. Breastfeeding (N=567, 56.3%)**	**2.Mixed milk feeding (N=102, 10.1%)**	**3. No breastfeeding (N=339, 33.6%)**	**Pairwise comparisons**			
				**p-value**	**1 vs 2**	**1 vs 3**	**2 vs 3**
**General characteristics**							
Age (years)	32.4 ± 4.1	32.5 ± 5.1	31.9 ± 4.5	0.230	0.853	0.100	0.287
% Non-Caucasian	19.3 (109)	32.3 (33)	8.3 (28)	**<.001**	**0.003**	**<.001**	**<.001**
Non-Caucasian:				**<.001**	**0.008**	**0.003**	**<.001**
% Asian	6.5 (37)	7.8 (8)	2.4 (8)				
% Northern African	5.8 (33)	6.9 (7)	2.4 (8)				
% Turkish	1.4 (8)	1.0 (1)	0.6 (2)				
% Subsaharan African	2.2 (12)	6.9 (7)	1.2 (4)				
% Middle East	1.4 (8)	5.9 (6)	0.6 (2)				
% Latin American	0.9 (5)	2.0 (2)	0.9 (2)				
% Other	1.1 (6)	2.0 (2)	0.3 (3)				
% Higher degree diploma	83.1 (466)	67.0 (67)	62.0 (206)	**<.001**	**<.001**	**<.001**	0.368
Highest education				**<.001**	**<.001**	**<.001**	0.626
% None/primary school	0.9 (5)	3.9 (4)	2.4 (8)				
% Lower secondary school	5.8 (33)	10.8 (11)	13.7 (46)				
% Higher secondary school	10.6 (60)	19.6 (20)	22.6 (76)				
% Higher education	82.6 (466)	65.7 (67)	61.3 (206)				
% Paid professional activity	89.7 (507)	80.4 (82)	90.2 (305)	**0.014**	**0.007**	0.808	**0.007**
Profession				0.171	0.064	0.542	0.165
% Employee	59.0 (334)	50.0 (51)	56.4 (190)				
% Laborer	8.8 (50)	10.8 (11)	8.6 (29)				
% Official	14.3 (81)	9.8 (10)	14.2 (48)				
% Self-employed	7.1 (40)	10.8 (11)	10.4 (35)				
% Other	10.8 (61)	18.6 (19)	10.4 (35)				
Monthly net income family				**<.001**	**0.016**	**0.004**	0.056
%<€1500	3.7 (21)	8.9 (9)	3.2 (11)				
%€1500-5000	81.2 (457)	83.2 (84)	89.0 (301)				
% >€5000	15.1 (85)	7.9 (8)	7.7 (26)				
% Living without partner	12.5 (71)	15.8 (16)	18.1 (61)	0.071	0.365	**0.022**	0.601
% History of smoking	24.3 (134)	30.0 (30)	29.6 (90)	0.171	0.225	0.090	0.940
**Medical history**							
% Multiparity	49.4 (280)	43.1 (44)	49.6 (168)	0.481	0.245	0.959	0.255
% First degree family history of T2DM	26.1 (142)	29.9 (29)	23.6 (76)	0.428	0.443	0.404	0.210
% Second degree family history of T2DM	58.0 (279)	59.8 (52)	55.9 (151)	0.776	0.759	0.581	0.529
% History of GDM	19.5 (68)	13.5 (7)	18.7 (39)	0.583	0.299	0.811	0.379
% History of PCOS	5.1 (28)	5.0 (5)	3.4 (11)	0.503	0.970	0.248	0.469
% History of miscarriage	33.7 (191)	28.4 (29)	32.1 (109)	0.565	0.298	0.635	0.477
Pre-pregnancy weight (kg)	68.9 ± 14.8	66.2 ± 12.9	74.8 ± 16.1	0.106	0.631	0.072	0.059
Pre-pregnancy BMI (kg/m²)	25.6 ± 5.0	26.7 ± 5.7	26.8 ± 5.5	**0.005**	0.097	**0.002**	0.766
Pre-pregnancy BMI classes				0.144	0.375	**0.032**	0.928
% Underweight (BMI < 18.5)	2.1 (12)	1.0 (1)	2.6 (9)				
% Normal (BMI 18.5-24.9)	49.9 (282)	43.4 (44)	43.4 (147)				
% Overweight (BMI 25.0-29.9)	29.6 (167)	28.4 (29)	26.2 (89)				
% Obese class 1 (BMI 30-34.9)	12.4 (70)	19.6 (20)	18.6 (63)				
% Obese class 2 (BMI 35-39.9)	5.5 (31)	6.9 (7)	7.7 (26)				
% Obese class 3 (BMI ≥ 40)	0.5 (3)	1.0 (1)	1.5 (5)				
**Delivery data maternal outcomes**							
Gestational age (weeks)	38.4 ± 1.6	38.4 ± 1.7	38.3 ± 1.4	0.091	0.665	**0.046**	0.115
Maternal weight at delivery	78.9 ± 13.7	81.7 ± 15.7	83.3 ± 15.5	**<.001**	0.121	**<.001**	0.293

Gestational weight gain (kg)	8.6 ± 5.2	9.9 ± 7.1	9.2 ± 6.0	0.068	**0.022**	0.298	0.149
% Inadequate weight gain	56.1 (293)	45.4 (45)	46.7 (147)	**0.012**	0.051	**0.008**	0.833
% Excessive weight gain	12.8 (67)	24.2 (24)	22.5 (71)	**<.001**	**0.003**	**<.001**	0.725
Type of labor<br>% Spontaneous<br>% Induced<br>% Caesarean section before labor	 43.21 (245)<br>46.38 (263)<br>10.41 (59)	 42.16 (43)<br>42.16 (43)<br>15.69 (16)	 40.41 (137)<br>43.36 (147)<br>16.22 (55)	0.122	0.285	**0.038**	0.952
**Delivery data neonatal outcomes**							
Birth weight (g)	3293.46 ± 484.160	3216.85 ± 520.837	3286.28 ± 484.923	0.411	0.219	0.428	0.491
Birth length (cm)	50.38 ± 2.068	48.93 ± 2.337	50.41 ± 1.831	0.122	**0.045**	0.950	0.070
% Macrosomia (> 4000g)	5.64 (32)	3.92 (4)	7.08 (24)	0.445	0.478	0.385	0.251
% Birth weight > 4500g	0.00 (0)	0.98 (1)	0.88 (3)	0.075	**0.018**	**0.025**	0.929
% LGA	11.46 (65)	7.84 (8)	12.39 (42)	0.448	0.280	0.676	0.204
% SGA	4.41 (25)	11.76 (12)	5.31 (18)	**0.011**	**0.003**	0.537	**0.023**
% NICU admission	24.29 (34)	40.63 (13)	28.21 (22)	0.174	0.061	0.525	0.204
Days on NICU	16.45 ± 18.063	12.83 ± 17.372	5.70 ± 4.943	0.122	0.589	**0.037**	0.367

T2DM: type 2 diabetes mellitus; GDM: gestational diabetes mellitus; PCOS: polycystic ovary syndrome; BMI: body mass index; LGA: Large for Gestational Age; SGA: Small for Gestational Age; NICU: Neonatal Intensive Care Unit. Categorical variables are presented as frequencies % (n); continuous variables are presented as mean ± SD if normally distributed and as median ± IQR if not normally distributed; Differences are considered significant at p-value<0.05. Bold means a statistical significant value of p<0.05.

Supplementary Table II. Participant postpartum characteristics according to breastfeeding behavior in the BEDIP-N and MEDLINA studies

Total cohort (N=1008)							
	**1. Breastfeeding (N=567, 56.3%)**	**2.Mixed milk feeding (N=102, 10.1%)**	**3. No breastfeeding (N=339, 33.6%)**	**Pairwise comparisons**			
				**p-value**	**1 vs 2**	**1 vs 3**	**2 vs 3**
**Postpartum**							
Breastfeeding duration				**<.001**	0.571	NA	NA
0-1 month	0.3 (2)	1.0 (1)	NA				
1-2 months	11.7 (66)	7.8 (8)					
2-3 months	40.4 (228)	42.7 (43)					
3-4 months	47.6 (269)	49.0 (50)					
Mean breastfeeding duration (months)	3.8 ± 2.4	3.7 ± 2.1	NA	0.635	0.492	NA	NA
% glucose intolerance	22.3 (126)	25.5 (26)	29.5 (100)	**0.004**	0.098	**0.019**	**0.011**
% T2DM	1.6 (9)	4.9 (5)	0.9 (3)	**<.001**	**0.004**	**<.001**	0.054
% IFG	3.7 (21)	9.8 (10)	11.8 (40)				
% IGT	15.2 (86)	7.8 (8)	12.7 (43)				
% IFG+IGT	1.8 (10)	3.0 (3)	4.1 (14)				
% Use of contraception	60.8 (345)	53.9 (55)	67.0 (227)	**0.035**	0.189	0.065	**0.016**
Use of contraception							
% Abstinention	2.1 (12)	1.0 (1)	0.9 (3)	0.312	0.444	0.160	0.929
% Condom	15.0 (85)	8.8 (9)	8.8 (30)	**0.013**	0.099	**0.007**	0.994
% Combined pill	1.2 (7)	0.0 (0)	33.6 (114)	**<.001**	0.259	**<.001**	**<.001**
% POP	37.7 (214)	36.3 (37)	9.4 (32)	**<.001**	0.778	**<.001**	**<.001**
% Levonorgestrel IUD	5.1 (29)	9.8 (10)	10.6 (36)	**0.006**	0.063	**0.002**	0.813
% Copper IUD	1.8 (10)	2.0 (2)	2.4 (8)	0.824	0.890	0.534	0.812
% Other	1.2 (7)	0.0 (0)	3.6 (12)	**0.016**	0.259	**0.019**	0.054
Timing OGTT (weeks)	11.9 ± 3.4	12.4 ± 3.7	12.0 ± 3.3	0.407	0.213	0.443	0.461
Weight mother (kg)	70.2 ± 13.7	73.8 ± 15.8	75.5 ± 15.8	**<.001**	**0.025**	**<.001**	0.338
BMI (kg/m²)	25.5 ± 4.7	27.3 ± 5.7	27.4 ± 5.6	**<.001**	**0.001**	**<.001**	0.972
BMI classes				**<.001**	**0.001**	**<.001**	0.733
% Underweight (BMI < 18.5)	2.0 (11)	2.0 (2)	2.4 (8)				
% Normal (BMI 18.5-24.9)	51.4 (290)	38.2 (39)	37.3 (126)				
% Overweight (BMI 25.0-29.9)	30.7 (173)	25.5 (26)	32.3 (109)				

% Obese class 1 (BMI 30-34.9)<br>% Obese class 2 (BMI 35-39.9)<br>% Obese class 3 (BMI ≥ 40)	11.4 (64)<br>4.1 (23)<br>0.5 (3)	23.6 (24)<br>8.8 (9)<br>2.0 (2)	17.5 (59)<br>8.6 (29)<br>2.1 (7)				
% Normal/underweight<br>% Overweight (BMI 25.0-29.9)<br>% Obese (BMI ≥ 30)	53.4 (301)<br>30.7 (173)<br>16.0 (90)	40.2 (41)<br>25.5 (26)<br>34.3 (35)	39.6 (134)<br>32.3 (109)<br>28.1 (95)	**<.001**	**<.001**	**<.001**	0.334
% Obesity	16.0 (90)	34.3 (35)	28.1 (95)	**<.001**	**<.001**	**<.001**	0.228
Mean systolic blood pressure (mmHg)	115.8 ± 11.4	115.7 ± 13.7	120.8 ± 12.5	**<.001**	0.908	**<.001**	**<.001**
Mean diastolic blood pressure (mmHg)	74.0 ± 9.3	74.0 ± 10.2	76.2 ± 9.9	**0.002**	0.926	**<.001**	**0.039**
% Hypertension (systolic BP ≥ 140 and/or diastolic BP ≥ 90 mmHg)	6.4 (36)	8.8 (9)	8.6 (29)	0.401	0.371	0.218	0.945
Waist circumference (cm)	88.0 ± 11.6	91.4 ± 12.0	92.2 ± 12.6	**<.001**	**0.003**	**<.001**	0.767
Waist circumference<br>% < 80cm<br>% 80-88 cm<br>% >88 cm	 23.1 (127)<br>31.6 (174)<br>45.3 (249)	 16.8 (17)<br>23.8 (24)<br>59.4 (60)	 17.3 (57)<br>23.3 (77)<br>59.4 (196)	**<.001**	**0.033**	**<.001**	0.993
% Waist circumference ≥ 80 cm	76.9 (423)	83.2 (84)	82.7 (273)	0.074	0.164	**0.040**	0.918
% Waist circumference > 88 cm	46.6 (256)	60.4 (61)	59.7 (197)	**<.001**	**0.010**	**<.001**	0.900
PPWR (kg)	-0.3 ± 4.9	2.0 ± 5.7	1.6 ± 5.6	**<.001**	**<.001**	**<.001**	0.285
PPWR ≤ 0 kg<br>PPWR 0-5 kg<br>PWWR > 5 kg	52.4 (295)<br>35.9 (202)<br>11.7 (66)	30.4 (31)<br>50.0 (51)<br>19.6 (20)	39.6 (134)<br>40.2 (136)<br>20.1 (68)	**<.001**	**<.001**	**<.001**	0.169
% PPWR > 0 kg	47.6 (268)	69.6 (71)	60.4 (204)	**<.001**	**<.001**	**<.001**	0.091
% PPWR > 5 kg	11.6 (66)	19.6 (20)	20.1 (68)	**0.001**	**0.027**	**<.001**	0.920
HbA1c (%)	5.3 ± 0.3	5.4 ± 0.4	5.2 ± 0.3	**<.001**	**0.009**	**<.001**	**<.001**
Fasting total cholesterol (mg/dl)<br>Fasting HDL-cholesterol (mg/dl)<br>Fasting LDL-cholesterol (mg/dl)<br>Fasting Triglycerides (mg/dl)	187.2 ± 36.1<br>64.3 ± 14.5<br>111.8 ± 32.7<br>68.9 ± 31.5	190.6 ± 35.2<br>60.7 ± 14.6<br>112.9 ± 29.2<br>100.1 ± 96.6	185.4 ± 34.0<br>58.8 ± 15.2<br>110.6 ± 31.5<br>111.0 ± 51.3	0.634<br>**<.001**<br>0.818<br>**<.001**	0.399<br>**0.029**<br>0.643<br>**<.001**	0.816<br>**<.001**<br>0.761<br>**<.001**	0.346<br>0.160<br>0.527<br>**<.001**
FPG (mg/dl)<br>Glycemia 30 min (mg/dl)	86.1 ± 8.2<br>147.7 ± 25.2	90.4 ± 9.6<br>150.8 ± 28.9	91.1 ± 8.5<br>145.0 ± 25.3	**<.001**<br>0.131	**<.001**<br>0.345	**<.001**<br>0.152	0.254<br>0.054

Glycemia 60 min (mg/dl)	146.3 ± 36.7	142.8 ± 41.3	136.5 ± 33.9	**<.001**	0.138	**<.001**	0.339
Glycemia 120 min (mg/dl)	114.8 ± 30.7	114.9 ± 33.5	114.6 ± 30.1	0.797	0.510	0.839	0.591
% Glucose intolerance (prediabetes + diabetes)	22.3 (126)	25.5 (26)	29.5 (100)	**0.004**	0.098	**0.019**	**0.011**
Glucose intolerance group:				**<.001**	**0.004**	**<.001**	0.054
IFG	3.7 (21)	9.8 (10)	11.8 (40)				
IGT	15.2 (86)	7.8 (8)	12.7 (43)				
IFG+IGT	1.8 (10)	2.9 (3)	4.1 (14)				
T2DM	1.6 (9)	4.9 (5)	0.9 (3)				
Fasting insulin (pmol/l)	49.7 ± 33.4	67.6 ± 40.1	74.5 ± 45.4	**<.001**	**<.001**	**<.001**	0.124
Insulin 30 min (pmol/l)	382.0 ± 221.1	495.9 ± 319.1	489.7 ± 324.4	**<.001**	**<.001**	**<.001**	0.808
Insulin 60 min (pmol/l)	280.7 ± 375.5	589.0 ± 418.2	556.7 ± 371.1	**<.001**	**0.001**	**<.001**	0.678
Insulin 120 min (pmol/l)	341.9 ± 252.2	425.0 ± 267.8	487.5 ± 370.1	**<.001**	**<.001**	**<.001**	0.299
Matsuda insulin sensitivity	5.5 (3.8-7.6)	4.1 (2.7-6.2)	4.0 (2.6-5.5)	**<.001**	**<.001**	**<.001**	0.576
HOMA-IR	1.3 (0.9-1.9)	1.9 (1.1-2.9)	2.0 (1.4-3.1)	**<.001**	**<.001**	**0.001**	0.114
HOMA-B	98.3 (72.1-140.6)	115.5 (81.9-175.4)	120.8 (91.0-172.0)	**<.001**	**0.016**	**<.001**	0.205
ISSI-2	2.0 (1.6-2.6)	2.0 (1.5-2.7)	1.8 (1.4-2.4)	**0.010**	0.960	**0.003**	0.087
Insulinogenic index/ HOMA-IR	0.3 (0.2-0.4)	0.2 (0.1-0.4)	0.2 (0.1-0.3)	**<.001**	0.273	**<.001**	0.132
IPAQ METs category at time of OGTT				0.311	0.711	0.200	0.144
% Low	10.5 (59)	13.0 (13)	6.9 (23)				
% Moderate	45.5 (256)	46.0 (46)	47.1 (157)				
% High	44.0 (248)	41.0 (41)	45.9 (153)				
% IPAQ category low	11.8 (66)	10.8 (11)	9.2 (31)	0.482	0.772	0.227	0.633
% Clinical depression (≥16 on CES-D questionnaire)	15.0 (85)	24.5 (25)	19.8 (67)	**0.028**	**0.017**	0.063	0.301

POP: progestogen only pill; IUD: intra uterine device; OGTT: oral glucose tolerance test ; BMI: body mass index; PPWR; postpartum weight retention; HDL-cholesterol: high density lipoprotein cholesterol; LDL-cholesterol: low density lipoprotein cholesterol; FPG; fasting plasma glucose; HOMA-IR : Homeostasis Model of Assessment – Insulin Resistance; HOMA-B: Homeostasis Model of Assessment – Beta-cell Function; ISSI-2: insulin secretion-sensitivity index-2; IPAQ: International Physical Activity Questionnaire; METs: metabolic syndrome; CES-D: Center for Epidemiologic Studies Depression; NA: not applicable; T2DM: Type 2 Diabetes Mellitus; IFG: Impaired Fasting Glycemia; IGT: Impaired Glucose Tolerance. Categorical variables are presented as frequencies %(n); continuous variables are presented as mean ± SD if normally distributed and as median ± IQR if not normally distributed; Differences are considered significant at p-value<0.05. Bold means a statistical significant value of p<0.05.

Supplementary Table III. Comparison of general characteristics between group excluded for sub-analysis and group included in sub-analysis

	**Excluded**<br><br>**(N=415, 29.16%)**	**Included**<br><br>**(N=1008, 70.84%)**	**p-value**
**General characteristics**			
Age (years)	32.0 ± 4.4	32.2 ± 4.4	0.617
% Non-Caucasian	15.5 (64)	16.9 (170)	0.518
Non-Caucasian:<br>% Asian<br>% Northern African<br>% Turkish<br>% Subsaharan African<br>% Middle East<br>% Latin American<br>% Other	 6.3 (26)<br>3.2 (13)<br>1.0 (4)<br>2.0 (8)<br>1.2 (5)<br>0.5 (2)<br>1.5 (6)	 5.3 (53)<br>4.8 (48)<br>1.1 (11)<br>2.3 (23)<br>1.6 (16)<br>1.0 (10)<br>0.9 (9)	0.700
% Higher degree diploma	73.3 (293)	74.4 (739)	0.652
Highest education<br>% None/primary school<br>% Lower secondary school<br>% Higher secondary school<br>% Higher education	 2.0 (8)<br>8.9 (36)<br>17.0 (69)<br>72.2 (293)	 1.7 (17)<br>9.0 (90)<br>15.6 (156)<br>73.8 (739)	0.898
% Paid professional activity	87.4 (360)	89.0 (894)	0.398
Profession<br>% Employee<br>% Laborer<br>% Official<br>% Self-employed<br>% Other	 57.7 (237)<br>11.2 (46)<br>12.7 (52)<br>7.1 (29)<br>11.4 (47)	 57.2 (575)<br>9.0 (90)<br>13.8 (139)<br>8.6 (86)<br>11.4 (115)	0.620
Monthly net income family<br>%<€1500<br>%€1500-5000<br>% >€5000	 6.8 (28)<br>83.2 (341)<br>10.0 (41)	 4.1 (41)<br>84.0 (842)<br>11.9 (119)	0.067
% Living without partner	17.2 (71)	14.7 (148)	0.246
% History of smoking	31.4 (122)	26.6 (254)	0.071
% Multiparity	42.9 (178)	48.8 (492)	**0.042**
% First degree family history of T2DM	23.4 (93)	25.7 (247)	0.384
% Second degree family history of T2DM	56.6 (192)	57.5 (482)	0.782
% History of GDM	17.2 (39)	18.7 (114)	0.616
% History of PCOS	4.4 (18)	4.5 (44)	0.946
% History of miscarriage	31.3 (130)	32.6 (329)	0.630
Pre-pregnancy weight (kg)	71.6 ± 16.7	70.3 ± 15.2	0.646
Pre-pregnancy BMI (kg/m²)	27.1 ± 5.7	26.1 ± 5.3	**0.005**
Pre-pregnancy BMI classes<br>% Underweight (BMI < 18.5)<br>% Normal (BMI 18.5-24.9)<br>% Overweight (BMI 25.0-29.9)<br>% Obese class 1 (BMI 30-34.9)<br>% Obese class 2 (BMI 35-39.9)<br>% Obese class 3 (BMI ≥ 40)	 2.2 (9)<br>41.0 (168)<br>27.8 (114)<br>18.5 (76)<br>8.8 (36)<br>1.7 (7)	 2.2 (22)<br>47.0 (473)<br>28.3 (285)<br>15.2 (153)<br>6.4 (64)<br>0.9 (9)	0.131

T2DM: type 2 diabetes mellitus; GDM: gestational diabetes mellitus; PCOS: polycystic ovary syndrome; BMI: body mass index. Categorical variables are presented as frequencies % (n); continuous variables are presented as mean ± SD if normally distributed and as median ± IQR if not normally distributed; Differences are considered significant at p-value<0.05. Bold means a statistical significant value of p<0.05.
